# STAFF PERSPECTIVES ON COUNTERING STAFF-TO-RESIDENT MISTREATMENT IN LONG-TERM CARE FACILITIES

**DOI:** 10.1093/geroni/igz038.2133

**Published:** 2019-11-08

**Authors:** Melanie Couture, Milaine Alarie, Sarita Israel

**Affiliations:** Centre for research and expertise in social gerontology, CIUSSS West-Central Montreal, Montreal, Quebec, Canada

## Abstract

In Canada, as well as in other countries, resident mistreatment is common in long-term care (LTC) facilities. In many situations, residents are mistreated by LTC staff. To address this problem, LTC facility managers and their employees must play an active role in the prevention as well as in the management of staff-to-resident mistreatment situations. However, it is still unclear what type of support they need to counter this type of mistreatment. Using an exploratory descriptive qualitative design, twenty-one managers and employees working in four different LTC facilities participated in semi-structured individual interviews. To allow participants to express themselves without risking self-incrimination or feeling pressured to report colleagues, vignettes depicting fictitious and common situations of staff-to-resident mistreatment were used as a conversation starter. Data analysis was performed using Miles, Huberman & Saldaña (2013) analytical method. Results show that participants think that staff-to-resident mistreatment is mainly caused by three staff characteristics: 1) not having the psychological profile to work in LTC facilities; 2) lack of training; and/or 3) being overworked. Consequently, participants believe that mistreatment prevention starts by improving employee selection practices to ensure candidates have adequate attitudes and training to work in LTC facilities. They also argue that staff should receive more training regarding mistreatment. Lastly, support interventions are suggested to prevent and address situations involving staff experiencing high levels of stress for personal or work-related reasons. This study shows that both individual and organisational measures are needed to fight against staff-to-resident mistreatment in LTC facilities.

